# Phylogenetic studies of *Petaurista petauri* based on complete mitochondrial DNA sequences

**DOI:** 10.1080/23802359.2016.1225527

**Published:** 2016-09-18

**Authors:** Wei Wei, Wenliang Zhou, Qizhu Chen, Yumin Yang, Ali Krzton, Jie Hu, Dayong Li

**Affiliations:** a Key Laboratory of Southwest China Wildlife Resources Conservation (Ministry of Education), China West Normal University, Nanchong, China;; b Ministry of Education Key Laboratory for Biodiversity Science and Ecological Engineering, College of Life Sciences, Beijing Normal University, Beijing, China;; c Guangdong Province Key Laboratory for Biotechnology Drug Candicates, Guangdong Pharmaceutical University, Guangzhou, China;; d Soil and Fertilizer Institute, Sichuan Academy of Agricultural Science, Chengdu, China;; e School of Library and Information Science, Kent State University, Kent, OH, USA

**Keywords:** *Petaurista petauri*, mitochondrial genome, phylogenetic relationship, Bayesian inference

## Abstract

The giant flying squirrel *Petaurista petauri* is a large rodent studied by few researchers. Here, we sequenced the complete mitochondrial genome of *P. petauri*. Similar to the typical vertebrate mitochondrial genome, the mtDNA of *P. petauri* also contained 37 genes (13 protein-coding genes, 2 rRNA genes, and 22 tRNA genes) and a noncoding region (D-loop). We also analyzed the phylogenetic relationship of *P. Petauri* to 14 other closely related species using the Bayesian inference. This work will contribute to our understanding of this species’ evolution and conservation.

The giant flying squirrel *Petaurista petauri* (also known as *Petaurista yunnanensis*) is a large rodent distributed mainly in the southwest of China, and likely in Burma and Laos as well (Smith & Xie 2008). *Petaurista petauri* (family Sciuridae) is a solitary, nocturnal mammal that prefers to inhabit caves, rock tunnels, and other holes (Smith & Xie 2008). As no research has been done on the molecular evolution of this species, we know little of their phylogenetic history, and the taxonomy of this species remains controversial (Smith & Xie 2008). Currently, only three complete sequences of the mitochondrial DNA of genus *Petaurista* have been published (Kong et al. 2015). Given their uncertain taxonomy and remarkable subterranean lifestyle, sequencing the mitochondrial genome sequence of *P. petauri* was a priority. We hope this work will contribute to resolving the taxonomic dispute around *P. petauri* and provide more valuable information about their life history.

One adult *P. petauri* was collected from the wild in Yunnan, China (coordinates: E 99°18.639′, N 27°35.702′). This specimen is stored at a museum in the College of Life Sciences, China West Normal University, China, with accession No. PP-15-01. We used standard phenol–chloroform methods to extract the genomic DNA from the muscle tissue (Sambrook & Russell 2001). After obtaining the complete mitochondrial genome of *P. petauri*, sequences were uploaded to the GenBank database with accession number KX528208. The complete mtDNA of *P. petauri* is 16620bp in length. It contains 13 protein-coding genes (PCGs), 22 tRNA genes, and 2 rRNA genes for a total of 37 encoded genes, as well as a noncoding D-loop region. All genes were distributed on the H-strand except for the ND6 subunit gene and nine tRNA genes, which were encoded on the L-strand. Overall, the nucleotide base composition of the mtDNA is A (30.3%), G (14.7%), C (31.0%), and T (24.0%), indicating a strong AT bias (Shadel & Clayton 1997).

In order to resolve the taxonomic dispute surrounding *P. petauri* and understand its relationship to other species, we compared its mtDNA to that of 14 other species from the family Sciuridae to reconstruct the phylogenetic tree. Using jModelTest version 2.1.4 (Darriba et al. 2012), we evaluated several models for phylogenetic analysis of the nucleotide sequences. The best-fitting model was the General Time Reversible model (GTR) (Tavaré 1986) coupled with Gamma distributed with Invariant sites (G + I). The nucleotide sequences were used to generate a maximum-likelihood tree with bootstrapping (1000 replicates) using MEGA version 5 (Tamura et al. 2011). The phylogenetic tree is classified into two large clades and the outgroup (Figure 1). The first lineage, containing six genera (*Tamiops*, *Dremomys*, *Callosciurus*, *Marmota*, *Ictidomys*, and *Cynomys*), belongs to the subfamily Sciurinae. The second lineage, subfamily Pteromyinae, includes the genera *Hylopetes*, *Pteromys*, and *Petaurista.* The outgroup was *Rattus norvegicus*. We expect that this work can contribute to the molecular identification of *P. petauri* and be helpful to explore the phylogeny of the Sciuridae.

**Figure 1.**
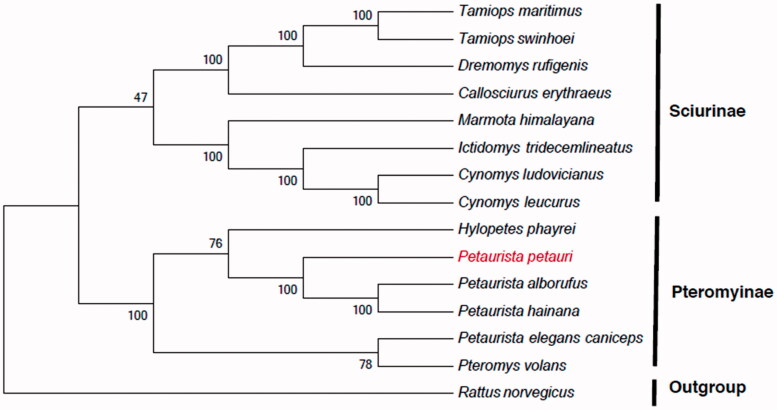
Maximum-likelihood tree inferred from the nucleotide sequence of whole mitogenome. The numbers above branches specify bootstrap percentages (1000 replicates). GenBank accession numbers for the published sequences are *H. phayrei* (NC026443); *P. volans* (JQ230001); *P. alborufus* (NC023922); *P. hainana* (JX572159); *P. elegans caniceps* (KU579289); *C. erythraeus* (NC025550); *C. leucurus* (NC026705); *C. ludovicianuse* (NC026706); *D. rufigenis* (NC026442); *I. tridecemlineatus* (NC027278); *M. himalayana* (NC018367); *T. maritimus* (NC029325); *T. swinhoei* (NC026875); *R. norvegicus* (KF011917).
